# Catalpol as a Component of *Rehmannia glutinosa* Protects Spinal Cord Injury by Inhibiting Endoplasmic Reticulum Stress-Mediated Neuronal Apoptosis

**DOI:** 10.3389/fphar.2022.860757

**Published:** 2022-07-08

**Authors:** Zhiyang Huang, Jiahong Gong, Wen Lin, Zhiyi Feng, Yirou Ma, Yurong Tu, Xiong Cai, Jianhua Liu, Chang Lv, Xinru Lv, Qiuji Wu, Wenjie Lu, Juan Zhao, Yibo Ying, Shengcun Li, Wenfei Ni, Haili Chen

**Affiliations:** ^1^ Rehabilitation Medicine Center, The Second Affiliated Hospital and Yuying Children’s Hospital of Wenzhou Medical University, Wenzhou, China; ^2^ Department of Orthopaedics, The Second Affiliated Hospital and Yuying Children’s Hospital of Wenzhou Medical University, Wenzhou, China; ^3^ School of Pharmaceutical Sciences, Wenzhou Medical University, Wenzhou, China; ^4^ Department of Physical Therapy, Beijing Bo’ai Hospital, China Rehabilitation Research Center, Beijing, China

**Keywords:** spinal cord injury, catalpol, neuroprotection, endoplasmic reticulum stress, apoptosis

## Abstract

Disturbance of the internal environment in the spinal cord after spinal cord injury (SCI) is an important cause of the massive death of neurons in the injury area and one of the major problems that lead to the difficult recovery of motor function in patients. *Rehmannia glutinosa*, a famous traditional Chinese medicine, is commonly used in neurodegenerative diseases, whereas an iridoid glycoside extract of catalpol (CAT), with antioxidant, antiapoptotic, and neuroprotective pharmacological effects. However, the neuroprotective and anti-apoptosis mechanism of CAT in SCI remains unclear. In our study, we found that CAT has a restorative effect on the lower limb motor function of rats with SCI by establishing a rat model of SCI and treating CAT gavage for 30 days. Our study further found that CAT has the effect of inhibiting apoptosis and protecting neurons, and the action pathway may reduce endoplasmic reticulum (ER) stress by inhibiting CHOP and GRP78 expression and then reduce apoptosis and protect neurons through the Caspase3/Bax/Bcl-2 pathway. In conclusion, we demonstrated that CAT can treat SCI by inhibiting ER stress-mediated neuronal apoptosis and has the potential to be a clinical drug for the treatment of SCI.

## Introduction

Spinal cord injury (SCI) is brought about via direct or indirect action of external forces on the spinal cord and a gradually increasing trend of incidence in the present society (Parent et al., 2011; Ahuja et al., 2017). It often leads to motor and sensory dysfunction of the lower limbs below the injured segment (Sauerbeck et al., 2021). Characterized by an exorbitant degree of morbidity, mortality, and cost, it drastically affects the healthy development of individuals, along with their families, and even society (Penas et al., 2007). Not only SCI can afflict directly on the spine by a mechanical impact but it also gives rise to severely secondary injury to the spinal cord which included disorders in ionic homeostasis, ischemia, focal hemorrhage, local edema, phlogistic response, and free radicals stress (Katoh et al., 1996; Wang et al., 2017; Fan et al., 2022). Nowadays, controlling and minimizing these secondary reactions have been the focus of surgical treatment. Many studies have proved that apoptosis plays a critical role in the secondary damage which causes progressive degeneration of the spinal cord (Yu and Fehlings, 2011; Li et al., 2019a). Therefore, treatment strategies designed to inhibit or delay apoptosis and cell death may contribute to the rehabilitation of SCI.

As early as 100 A.D., *Rehmannia glutinosa* (RG) was recorded in Shennong’s herbal classic as a plant of Scrophulariaceae. Rehmanniae Decoction of Six Ingredients has been indicated as a remedy for geriatric diseases for a long time, and its efficacy has been known (Wang et al., 2020). RG has also been used in traditional Chinese medicine (TCM) to treat aging-related conditions including dementia and senile diseases. At present, the iridoid glycosides are considered to be the main pharmacological components of RG, and we found that catalpol (CAT) has the best cytoprotective effect by screening the iridoid glycosides. Meanwhile, CAT is the most major component of the iridoid glycosides. It has been reported that CAT has anticancer and anti-inflammatory effects (Xia et al., 2020). It also can protect mesencephalic neurons from neurotoxic damage (Wang et al., 2019). In addition, CAT was found to enhance motor function recovery *via* increasing striatal dopamine concentration and glial cell line-derived neurotrophic factor level (Xu et al., 2010). Interestingly, the role of CAT in antiapoptosis has also been reported with neuroprotective effects against acute focal ischemic stroke (Wang et al., 2020). Because the death of neurons during SCI is the main reason for damaged motor function, we speculate that the neuroprotective effects of CAT can alleviate the neuronal death caused by SCI (Heller et al., 2021).

The apoptosis in SCI is related to the endoplasmic reticulum (ER) stress-induced action in neuronal cells (Han et al., 2013), which can be triggered by hypoglycemia, free radicals, and superimposition of proteins with abnormal folding (Wang and Kaufman, 2016). During ER stress, the expression of glucose-regulated protein 78 (GRP78), the activation of ER-associated Caspase-12, and the transcription activation of the C/EBP homologous transcription factor (CHOP) are all relevant to cell death. In SCI, excessive or abnormal ER stress leads to injury or death of neuronal cells by accumulating misfolded proteins in the ER lumen (inhibiting ER function) (Ohashi et al., 2016) and activating CHOP and Caspase-12 (resulting in secondary injury) (Myers et al., 2019; Zhu et al., 2019). It has been confirmed that ER stress plays a role in the apoptosis of neurons and oligodendrocytes after SCI but not in astrocytes (Ding et al., 2012; Liu et al., 2012). Thus, many therapeutic measures aim to target ER stress to inhibit apoptotic cell death and secondary damage after SCI.

In this experiment, we fed 30 days SCI rats with Rehmannia decoction, observed the recovery of motor function of rats, and found that CAT played a major pharmacological role in it. By further observing the recovery effect of CAT on SCI, the results showed that CAT can inhibit ER stress-mediated neuronal apoptosis in SCI and promote functional recovery in rats with SCI.

## Materials and Methods

### Reagents and Antibodies

CAT and verbascoside (VER) were provided by (MedChemExpress United States) with a purity of 98.04% and 99.83%, leonuride (LEO) was provided by Topscience (China) with a purity of 99.15%, and the product was tested and complied with the given specifications. Dulbecco’s modified Eagle’s medium and fetal bovine serum (FBS) were supplied by Invitrogen (Carlsbad, CA, United States). Primary antibodies including NeuN (ab104224), CHOP (ab11419), GRP78 (ab21685), GAP43 (ab78810), Caspase-12 (ab62484), and MAP-2 (ab5392) were supplied by Abcam (Cambridge, United Kingdom). Bax (R22708), Bcl-2 (250198), Caspase3 (383315), and Cleaved-Caspase3 (341034) were supplied by Zenbio (Chengdu, China). The second antibody antirabbit IgG-conjugated Alexa Fluor488 (ab150061), Alexa Fluor647 (ab150063), Alexa Fluor595 (ab150062), and second antibody antimouse IgG-conjugated Alexa Fluor488 (ab150109) were supplied by Abcam (Cambridge, United Kingdom). An enhanced chemiluminescence kit was supplied by Bio-Rad (Hercules, CA, United States).

### Animals

Eight-week-old female rats used in this experiment were provided by the animal experimental center of Wenzhou Medical University, Zhejiang, China. Before the experiment, the animals were maintained in a thermostatic room with a day/night cycle for 30 days. The animal experiment was conducted in full accordance with the guide for the Care and Use of Laboratory Animals.

### Experimental Design

Rats were randomly divided into Sham group, SCI group, and SCI + RG group with six rats in each group. Rats were anesthetized with an intraperitoneal injection of 1% pentobarbital sodium (0.35 ml/kg). Animals were placed on a cork platform and a partial laminectomy was performed at the T9 segment vertebral body to expose the vertebral column. Afterward, a vascular clip was inserted into the spinal cord for 1.5 min (Senbokuya et al., 2019), causing a moderate contusion injury (30 g force, Oscar, China). Animals in the control group underwent the same surgery but without injury to the spinal cord.

Assisting animals in urination twice daily from the date of SCI generation to the day of sacrificing the animals, rats were gavaged daily with Rehmannia decoction. The control group was treated with sodium chloride. After treatment, animals were monitored consistently until the final data analysis. Animals were subjected to two passive hind leg activities per day for rehabilitation. Thirty days after SCI, the rats were observed for hindlimb motor function recovery, and the lower limb motor ability was scored. Rats were humanely sacrificed by intraperitoneal injection of 1% pentobarbital sodium (0.35 ml/kg) into the rats.

In another set of experiments, rats were randomly divided into the Sham group, SCI group, SCI + CAT group with six rats in each group. The above modeling and animal handling steps were repeated. Rats were gavaged daily with 2 mg/rat of CAT from the day of SCI until the day animals were sacrificed. Rats were humanely sacrificed 30 days after SCI to obtain spinal cord tissue for subsequent experiments.

### Cell Culture and Treatment

Rat pheochromocytoma PC12 cells (American Type Culture Collection) were cultured in a complete medium (RPMI Medium 1,640 basic, Gibco, United States) containing 10% FBS (Invitrogen, Carlsbad, CA, United States), 100 units/mL penicillin and 100 μg/mL streptomycin. PC12 cells were incubated at 37°C with 5% CO_2_ in a cell culture incubator. PC-12 cell injury model was prepared with hydrogen peroxide, and PC-12 cells without any treatment were used as the control group.

### *In Vitro* Scratch Assay

Linear scratches in the cells were made by 200 uL pipette tips to have cell-free areas created. Thapsigargin (TG) was used as an inducer of ER stress, and 4-phenylbutyric acid (4-PBA) was used as an inhibitor of ER stress. After drug administration experiments, cells were washed with PBS twice. The blank areas were photographed and analyzed using an inverted microscope.

### CCK-8 Experiment

Cells were seeded in 96 well plates and then tested via different concentrations of medicine and hydrogen peroxide. Before being treated with H_2_O_2_ for 2 h, the cells were pretreated with medicine for 12 h. After that, 10 μL CCK-8 (Beyotime, Shanghai, China) was added to have absorbance measured via MX190.

### Flow Cytometry Analysis

Cells treated with CAT, H_2_O_2_, and H_2_O_2_ + CAT were processed for sample analysis using an Annexin V-FITC/PI apoptosis kit (MULTI SCIENCES, Shanghai, China) and then analyzed using a FACScan flow cytometer (Beckman Coulter, Cytoflex, United States).

### Locomotion Recovery Assessment

To have recovery conditions assessed, the physical conditions and ethological analyses of the rats were monitored by two individuals who were trained with the standard scoring criterion. The BBB locomotion scale was used to evaluate the movement function of the hindlimb with a scoring scale ranging from 0 to 21 (Basso et al., 1995). The inclined plane test was performed by placing the rat on top of an inclined plate with a rubber that is 6 mm thick, then the inclined angle was measured (Rivlin and Tator, 1977). In the footprint test, the rats’ hind paws were dipped with red dye to have the locomotor function analyzed as previously described (de Medinaceli et al., 1982). The following parameters were applied to evaluate locomotion function: 1) weight support, 2) leg extensor spasms, 3) the number of footsteps, and 4) the posture of the foot (Zhu et al., 2020a).

### TUNEL Apoptosis Assay

A second anesthetization was performed with 1% pentobarbital, which was terminated by thoracotomy on day 30. The spinal cord around the lesion was resected, and the central part of the injured area was fixed with cold 4% paraformaldehyde. Paraffin embedding was performed, and paraffin sections were obtained. TUNEL Apoptosis Detection Kit (YEASEN Biotech, Shanghai, China) was used to detect apoptosis in spinal cord lesions, and TUNEL-positive cells were observed under a Nikon ECLIPSE 80i (Nikon, Japan) at a magnification of 4×.

### Hematoxylin–Eosin Staining and Immunohistochemistry

HE staining was performed using spinal cord paraffin sections. The slides were incubated in 1% cresyl violet for Nissl staining, then observed and photographed under a light microscope.

In immunohistochemistry assays, spinal cord tissue was incubated in a blocking solution that contained milk (5%) and serum (3%) in PBS−0.1% Triton TM X-100 (Sigma) for 1 h. The slides were treated with primary antibodies at 4°C overnight and then treated with corresponding secondary antibodies conjugated by horseradish peroxidase. The immune reaction was carried out with 3, 3-diaminobenzidine staining. A positive signal in cells was photographed using a Nikon ECLIPSE 80i (Nikon, Japan) at a magnification of ×40. The statistics of optical densities in positive signals were performed at three picked fields per sample.

### Immunofluorescence Staining

At ambient temperature, the sections were treated with 5% BSA in PBS containing 0.1% Triton X-100 for 1 h and then incubated with different primary antibodies at 4°C in the same buffer overnight. NeuN (1:300), GAP43 (1:300), MAP-2 (1:300) and Caspase3 (1:300), Caspase12 (1:300), GRP78 (1:300), and C-Caspase3 (1:300) were used as primary antibodies. After that, the tissue was treated with a corresponding secondary antibody for 1 h at 37°C. The nuclei were stained with DAPI. Every image was captured using a Nikon ECLIPSE Ti microscope (Nikon, Tokyo, Japan). ImageJ (version 1.8.0) was used to analyze the results.

### Western Blot

Tissue obtained from the central part of the SCI area was frozen at −80°C for subsequent Western blot analysis. Cell lysates containing phenylmethanesulfonyl fluoride and phosphatase inhibitors were homogenized. The homogenate was then centrifuged to have supernatant collected. The concentration of protein was quantified using Coomassie blue. Gels were loaded per lane with 40 μg protein and then transferred onto polyvinylidene fluoride membranes (bio-rad, Hercules, CA, United States). Membranes were incubated with a rapid blocking solution (Beyotime, Shanghai, China) for 20 min. The samples were treated with primary antibodies overnight at 4°C including Caspase3 (1:1000), C-Caspase3 (1:1000), CHOP (1:1000), Caspase12 (1:1000), GRP78 (1:1000), Bax (1:1000), Bcl-2 (1:1000), and GAPDH (1:5000), and then incubated with secondary antibodies at room temperature for 2 h. The signal was detected via ChemiDoc XRS + Imaging System (Bio-Rad). Multi-Gauge Software of Science Lab 2006 (FUJIFILM Corporation, Tokyo, Japan) was used to measure the bands. ImageJ (version 1.8.0) was used to quantitatively analyze the bands.

### Statistical Analysis

Different results between the experimental groups and the control groups were determined as the mean SD. Student’s t test was used to analyze the statistical significance of the two experimental groups. A two-way analysis of variance (ANOVA) test was used for statistical evaluation of data over two groups, and then Dunnett’s post hoc test was used. *p* < 0.05 was significant.

## Results

### Exploration of the Basic Situation of *Rehmannia glutinosa*

**FIGURE 1:**
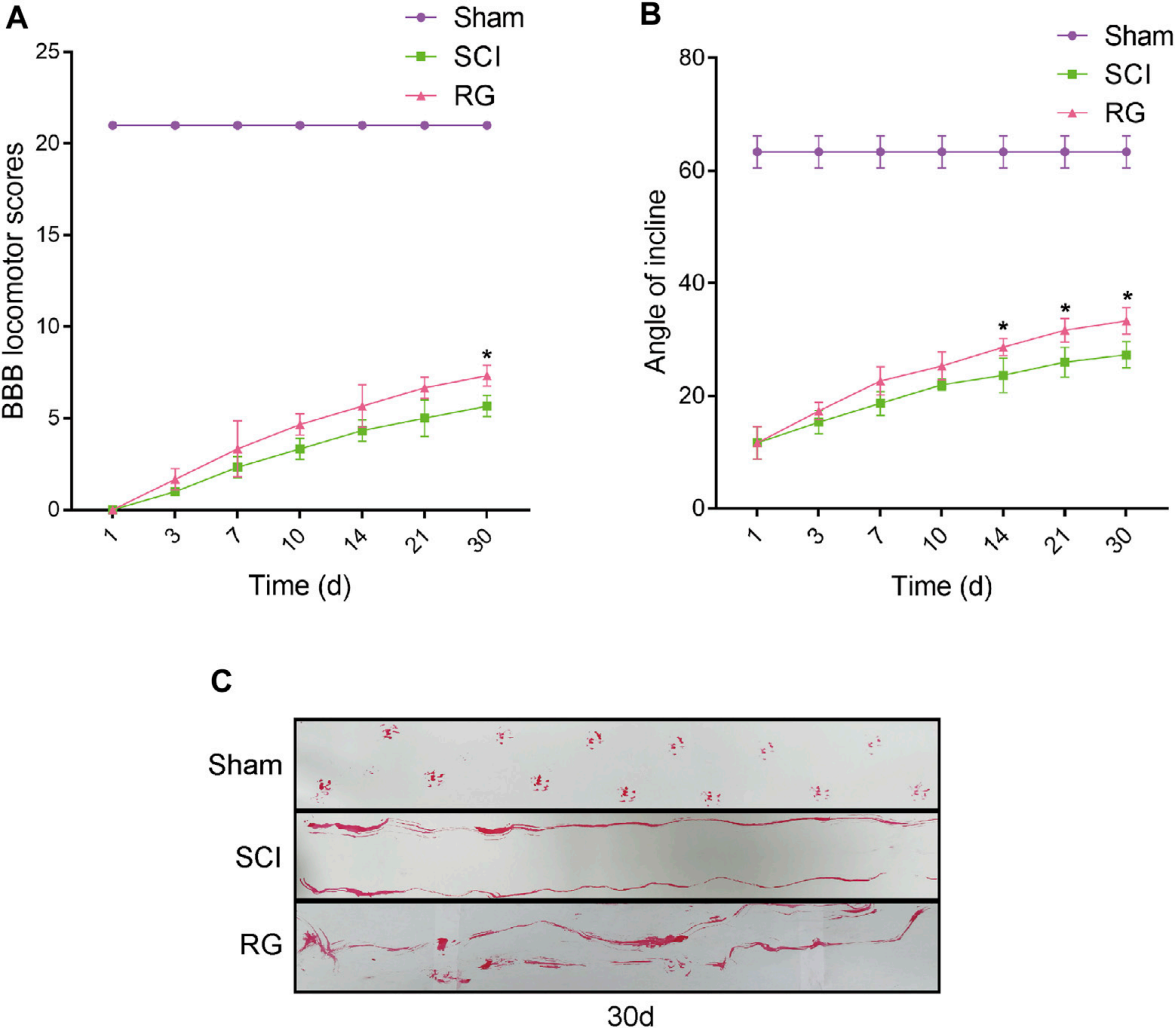
RG can slightly promote recovery from SCI in rats. **(A)** The BBB scores of the rats in the Sham, SCI, and RG groups. **(B)** The inclined plane test scores of rats in the Sham, SCI, and RG groups. **(C)** Gait imprint analysis. A two-way analysis of variance (ANOVA) test was used to analyze BBB scores and inclined plane test scores, and then Dunnett’s post hoc test was used. “*” represents *p* < 0.05 versus the SCI group with statistical significance. Data are represented as mean ± SD (*n* = 3).

The T9 SCI rats were fed Rehmannia decoction daily to observe their recovery. To examine the recovery of locomotor function, we assessed the locomotor activity of the hindlimbs of rats using the BBB score, inclined board test, and footprinting. The BBB motor scores of the Sham, SCI, and RG groups were assessed at 1, 3, 7, 10, 14, 21, and 30 days post-surgery. According to the BBB score, the rats became paralyzed immediately after surgery, it was suggested that the motor function of rats was slightly recovered in the RG group, as quantified in respect of BBB scores (Figure 1A). The rats in the RG group were slightly adapted to higher angles (Figure 1B). Footprint test analysis in RG-treated rats at 30 days after SCI showed a slight improvement in hindlimb coordination and toe dragging (Figure 1C).

### Catalpol Has a Better Recovery Effect Among the Components of *Rehmannia glutinosa* and Can Promote Motor Function Recovery

**FIGURE 2:**
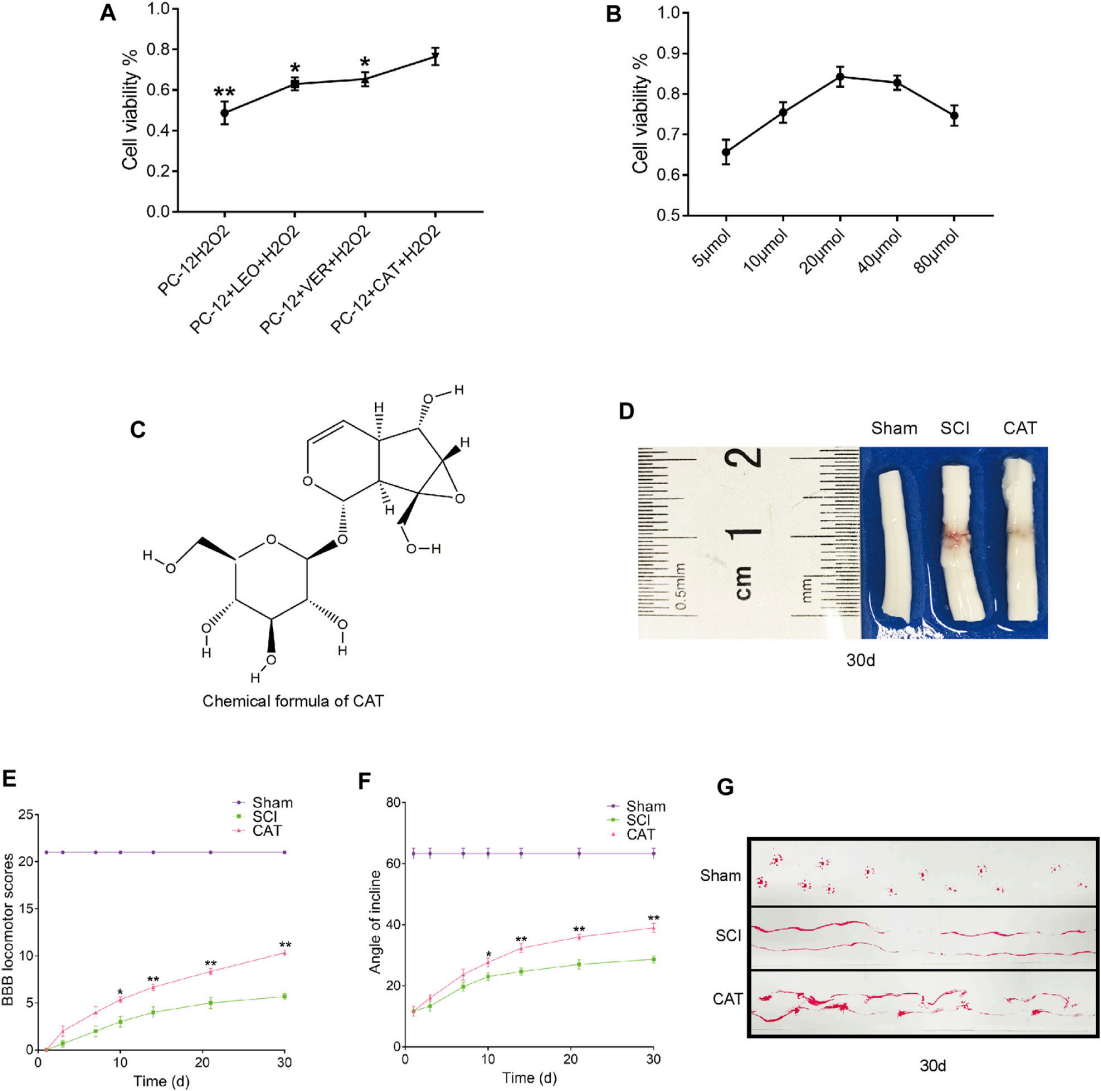
Screening against the therapeutic components of RG and found that CAT promotes locomotor function recovery in SCI rats. **(A)** CCK8 experiments were used to examine cell survival after LEO-, VER-, and CAT-protected cells from hydrogen peroxide stimulation. “*” represents *p* < 0.05, “**” represents *p* < 0.01 versus the PC-12 + CAT + H_2_O_2_ group. **(B)** Survival after different concentrations of CAT-protected cells in the face of hydrogen peroxide stimulation. **(C)** The chemical formula of CAT. **(D)** Spinal cord pictorial diagram. **(E,F)** The scores of BBB and the scores of the inclined plane by administration RG. **(G)** Gait imprint analysis. Student’s t test was used to analyze CCK8 experiments. A two-way analysis of variance (ANOVA) test was used to analyze BBB scores and inclined plane test scores, and then Dunnett’s post hoc test was used. “*” represents *p* < 0.05, “**” represents *p* < 0.01 versus the SCI group with statistical significance. Data are represented as mean ± SD (*n* = 3).

We first analyzed the components in RG and found that the recovery-promoting components were mainly iridoid glycosides. We then carried out a CCK-8 experiment on the main iridoid glycosides, VER, LEO, and CAT, to see whether they could resist hydrogen peroxide damage to PC-12 cells. We found that PC-12 cells, which were protected by CAT, could better resist the hydrogen peroxide stimulation (Figure 2A). Compared with the therapeutic effect of CAT, it can be inferred that CAT is the main component in RG to promote the recovery of SCI in rats. We further investigated the antiapoptotic effect of CAT. CAT at concentrations of 5–80 μmol showed protection when exposed to hydrogen peroxide stimulation in the face of PC-12, with 20 μmol showing the most significant protection (Figure 2B). The molecular formula of CAT (Figure 2C).

To observe the therapeutic effect of CAT *in vivo*, rats were administered orally for 30 days to observe the morphological changes in the spinal cord after SCI. Compared with the spinal cord of the SCI group, it was found that after CAT administration, the blood traces in the spinal cord of rats were reduced and the morphology was more perfect (Figure 2D). Evaluation of the quality of recovery of the animals’ functional condition by BBB locomotion scale and inclined plane test the results showed that after administration of CAT, the rats obtained a higher score, demonstrating that CAT had a better recovery effect (Figures 2E, F). Footprint test analysis of rats treated with CAT revealed a further reversal of hindlimb coordination and reduced toe dragging. By contrast, the footprints obtained from the vehicle-treated animals demonstrated inconsistent coordination and extensive toe dragging, as revealed by the ink streaks extending from both hindlimbs (Figure 2G).

### Positive Effect of Catalpol on Neurons

**FIGURE 3:**
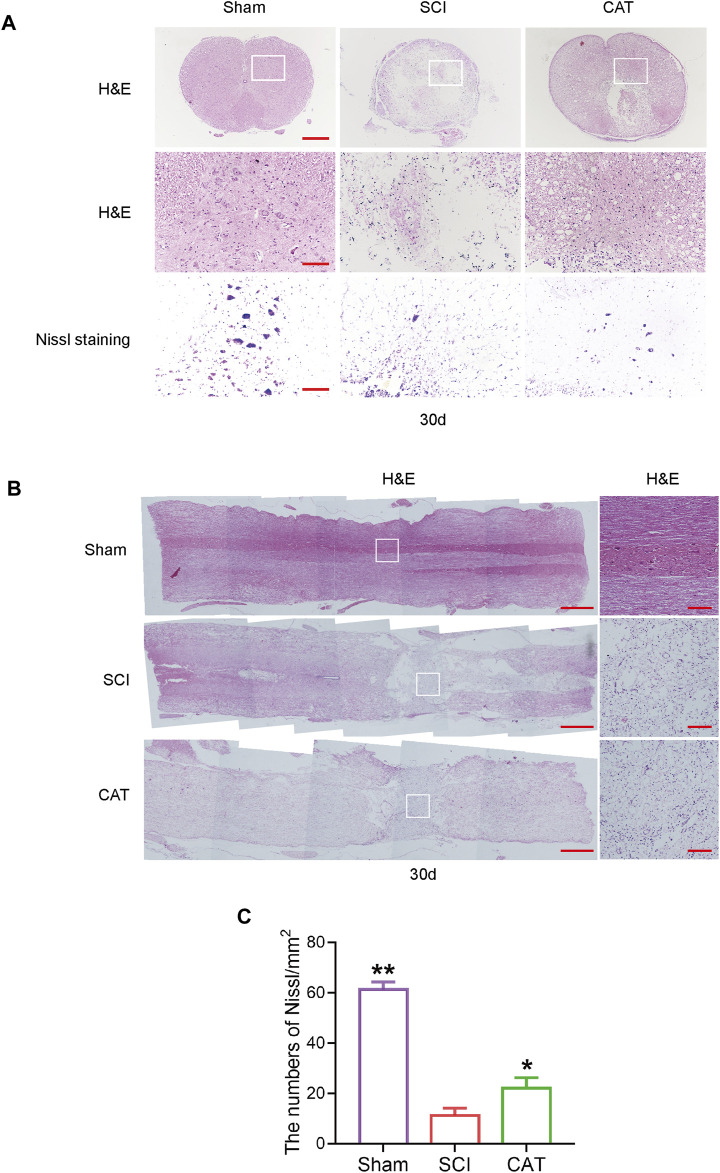
CAT improves the recovery of SCI rats. **(A)** H&E staining was performed on crosssection slides of spinal cords from the Sham group, the SCI group, and the CAT group, scale bar = 500 μm. Boxed areas are high-power image areas with scale bar = 100 μm. Nissl staining of samples from the Sham group, SCI group, and CAT group, scale bar = 100 μm. **(B)** Longitudinal sections of spinal cords from the Sham group, the SCI group, and the CAT group were subjected to H&E staining, scale bar = 500 μm. Boxed areas are high-power image areas, scale bar = 100 μm. **(C)** Analysis of the Nissl staining results. Student’s t test was used to analyze Nissl staining results. “*” represents *p* < 0.05, “**” represents *p* < 0.01 versus the SCI group with statistical significance. Data are represented as mean ± SD (*n* = 3).

To observe the protective effect of CAT on neurons, the cross and longitudinal sections of the spinal cord in each group were stained via H&E and Nissl staining (Figures 3A, B). Compared with the Sham group, the empty lacunae in the white and gray matter regions of the spinal cord were obvious in the SCI group on day 30, indicating that the injury was significantly aggravated. After CAT administration, a significant protective activity of less necrosis, karyopyknosis, and infiltrated polymorphonuclear leukocytes and macrophages was observed. In the SCI group, the number of large and medium-sized neurons in the intumescence spinal cord anterior horn of the lateral was less than in the Sham group. The survival rate of neurons was reduced, and the morphology of Nissl stained cells was vague and unclear and was recovered after CAT administration (Figures 3A, C). In conclusion, it was revealed by Nissl staining that the number of neurons in the anterior horn of the spinal cord was significantly higher in the CAT-treated group than in the vehicle-treated group, which indicated that CAT exerts neuroprotective effects in SCI rats.

### Catalpol Inhibits ER Stress-Induced Apoptosis in SCI Rats

**FIGURE 4:**
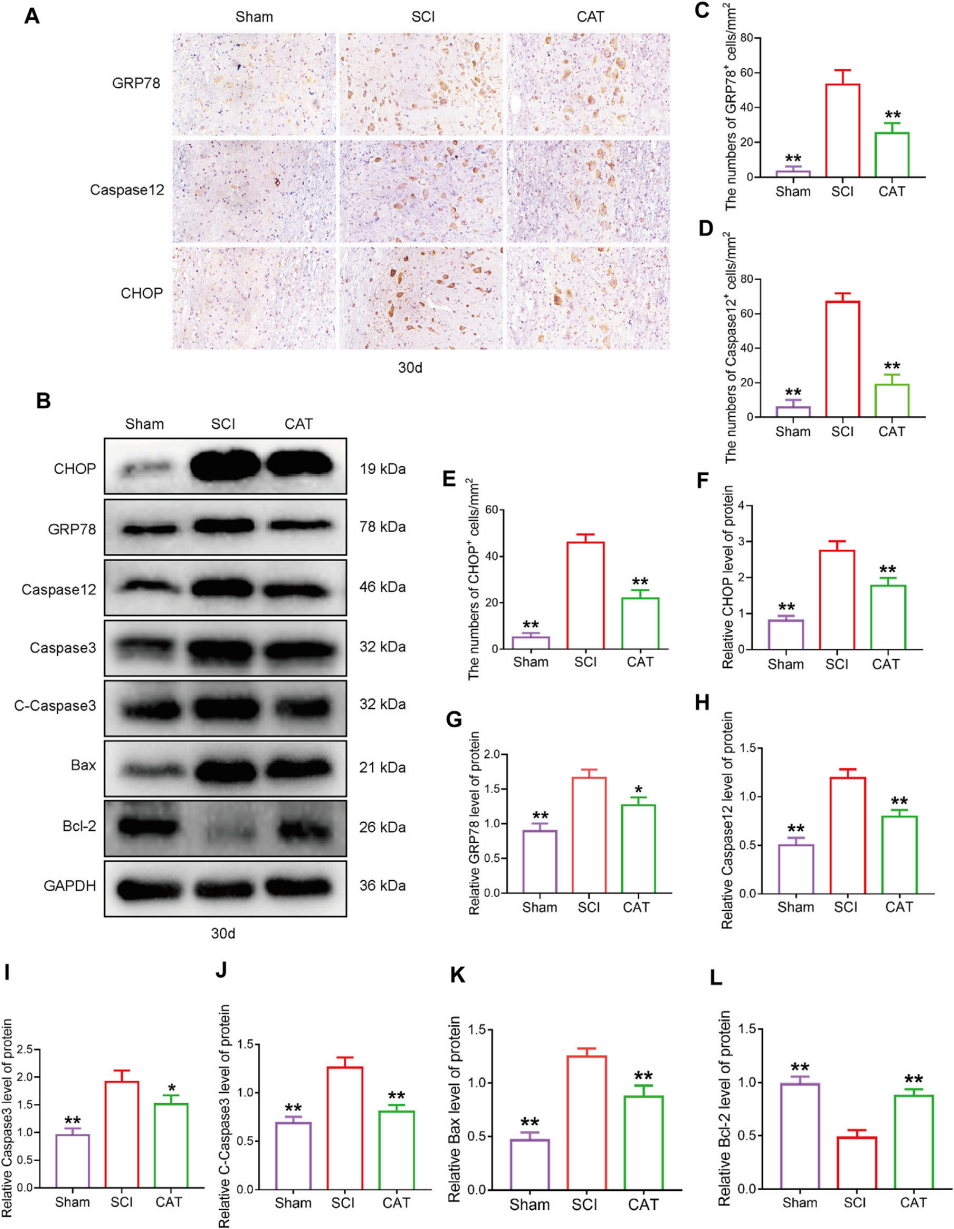
CAT inhibits ER stress-induced apoptosis in SCI rats. **(A)** Immunohistochemical test showing GRP78, CHOP, and Caspase-12 expression in the Sham group, SCI group, and CAT group. **(B)** Western blot analysis showing the protein expression of CHOP, GPR78, Caspase12, Caspase3, C-Caspase3, Bax, and Bcl-2 in the Sham group, SCI group, and CAT group. GAPDH is used as a loading control. **(C–E)** Analysis of optical density signals in immunostained cells. **(F–L)** Quantitative analysis of CHOP, GPR78, Caspase12, Caspase3, C-Caspase3, Bax, and Bcl-2 protein expression. Student’s t test was used to analyze Immunohistochemical and Western blot results. “*” represents *p* < 0.05, “**” *p* < 0.01 versus the SCI group with statistical significance. Data are represented as mean ± SD (*n* = 3).

To observe the expression of ER stress-induced apoptotic proteins after CAT treatment, we used immunohistochemistry to detect ER stress-related proteins. We found that the expression of GRP78, CHOP, and Caspase12 positive cells was more intense in the gray matter than in the white matter (Figure 4A). CAT treatment reduced the number of CHOP, GRP78, and Caspase-12 positive cells (Figures 4C–E). In Western blot analysis, compared with the SCI group, the CAT group showed decreased expression of CHOP and GRP78, evidenced by decreased ER stress, the expression of Caspase12, Caspase3, C-Caspase3, and Bax-related apoptosis promoting proteins, the expression trend, and the expression of Bcl-2 as an apoptosis inhibitory protein (Figures 4B, F–L). These data demonstrate that CAT has the effect of inhibiting ER stress to reduce apoptosis.

### Protective Effect of Catalpol on Neurons in SCI Rats

**FIGURE 5:**
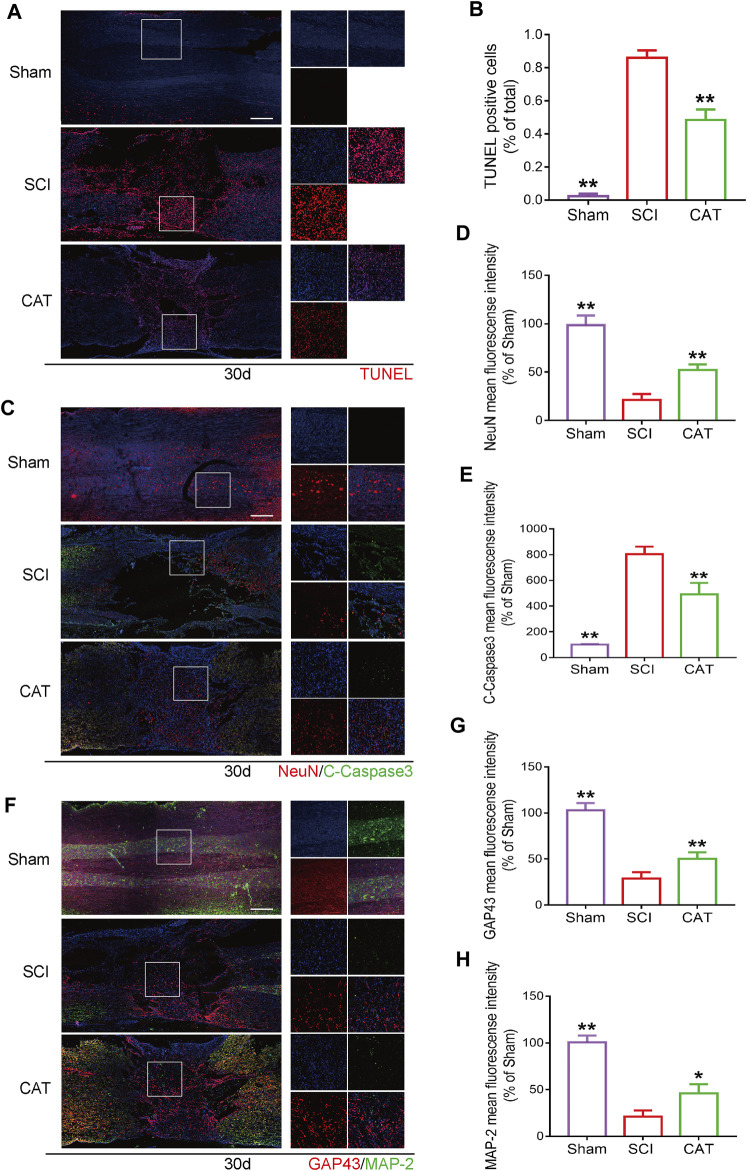
CAT inhibits neuron apoptosis and upregulates neuroprotective factors in SCI rats. **(A)** TUNEL staining of SCI area and magnification is ×4. **(B)** Analysis of the TUNEL staining results. **(C)** Immunofluorescence staining of NeuN (bright red) and C-Caspase-3 (bright green), which represents neurons in the Sham group, the SCI group, and the CAT group. **(F)** Immunofluorescence staining of GAP43 (bright red) and MAP-2 (bright green). The nuclei are labeled by DAPI (blue), and the magnification is ×4. **(D,E,G,H)** Quantitative analysis of immunofluorescence staining signals. Student’s t test was used to analyze immunofluorescence results. “*” represents *p* < 0.05, “**” represents *p* < 0.01 versus the SCI group with statistical significance. Data are represented as mean ± SD (*n* = 3).

The results of TUNEL staining to detect apoptotic cells in the spinal cord showed that compared with the SCI group, the TUNEL-positive cells in the spinal cord were decreased after CAT administration (Figures 5A, B). The surviving neurons after SCI are important for the recovery of motor function, and these neurons were detected with NeuN protein staining. It was revealed by immunofluorescence staining that the survival rate of neurons in the CAT administered group was higher than that in the SCI group, meanwhile, the expression of C-Caspase3 was also inhibited, and the results of the synergistic TUNEL staining (Figure 5A) could further demonstrate that the apoptosis of neurons in the injury area was inhibited in the CAT group (Figures 5C–E). The increased expression of GAP43 and MAP-2 (Figures 5F–H) indicated the enhanced growth and regeneration of neurons after CAT treatment, demonstrating that CAT has a role in promoting the repair of injured neurons. These results demonstrated that the neuroprotective effect of CAT on SCI rats was achieved by the upregulation of NeuN, GAP43, and MAP-2 proteins.

### Catalpol Protects the Inhibition of Cell Migration and Wound Repair of PC-12 Cells by Attenuating ER Stress

**FIGURE 6:**
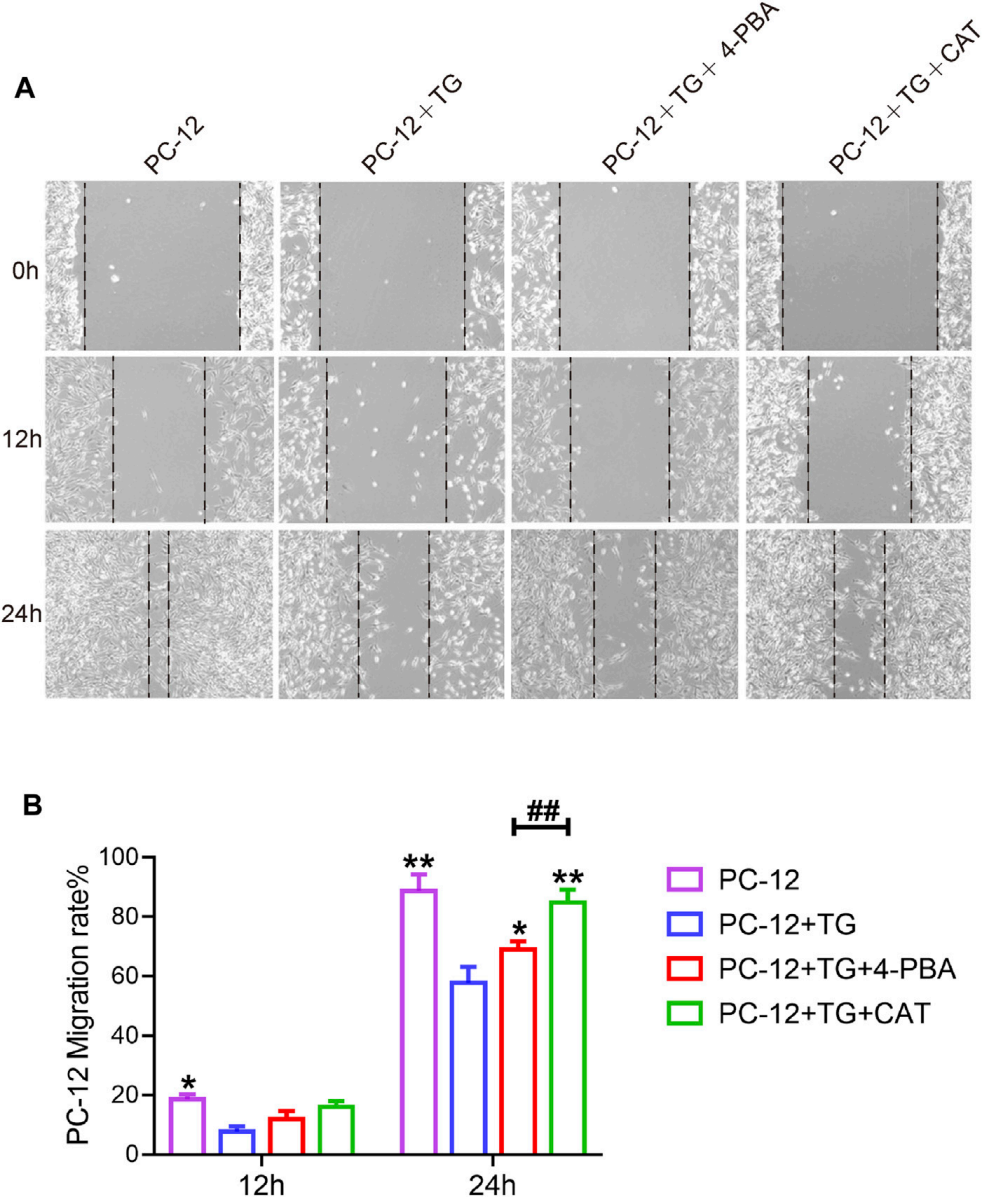
CAT promotes the cell migration of PC-12 cells. **(A)** Wound healing of the PC-12 group, PC-12 + TG group, PC-12 + TG + 4-PBA group, and PC-12 + TG + CAT group. All images were taken with an inverted phase-contrast microscope 0,12, and 24 h after cell scratches. **(B)** The migration rate of cells from the PC-12 group, PC-12 + TG group, PC-12 + TG + 4-PBA group, and PC-12 + TG + CAT group. A two-way analysis of variance (ANOVA) test was used to analyze cell migration results, and then Dunnett’s post hoc test was used. In the 12 h group, “*” represents *p* < 0.05 versus the PC-12 + TG group. In the 24 h group, “*” represents *p* < 0.05, “**” represents *p* < 0.01 versus the PC-12 + TG group, “##” represents *p* < 0.01 comparing the PC-12 + TG + 4-PBA group to the PC-12 + TG + CAT group with statistical significance. Data are represented as mean ± SD (*n* = 3).

PC12 cells have been shown to have similar functions to normal neuronal cells, such as cell morphology and physiological activities. To investigate whether CAT was able to promote cell migration and repair abilities by inhibiting ER stress. TG was used to trigger ER stress in PC12 cells, which was accompanied by inhibition of cell migration in a scratch wound assay. It was found that CAT-protected the inhibition of cell migration and wound repair of PC12 cells by TG-induced ER stress in a time course-dependent manner. As a positive control, 4-PBA was also found to protect the inhibition of cell migration via TG-triggered ER stress (Figures 6A, B).

### Catalpol Inhibits Hydrogen Peroxide-Induced Apoptosis of PC-12 Cells

**FIGURE 7:**
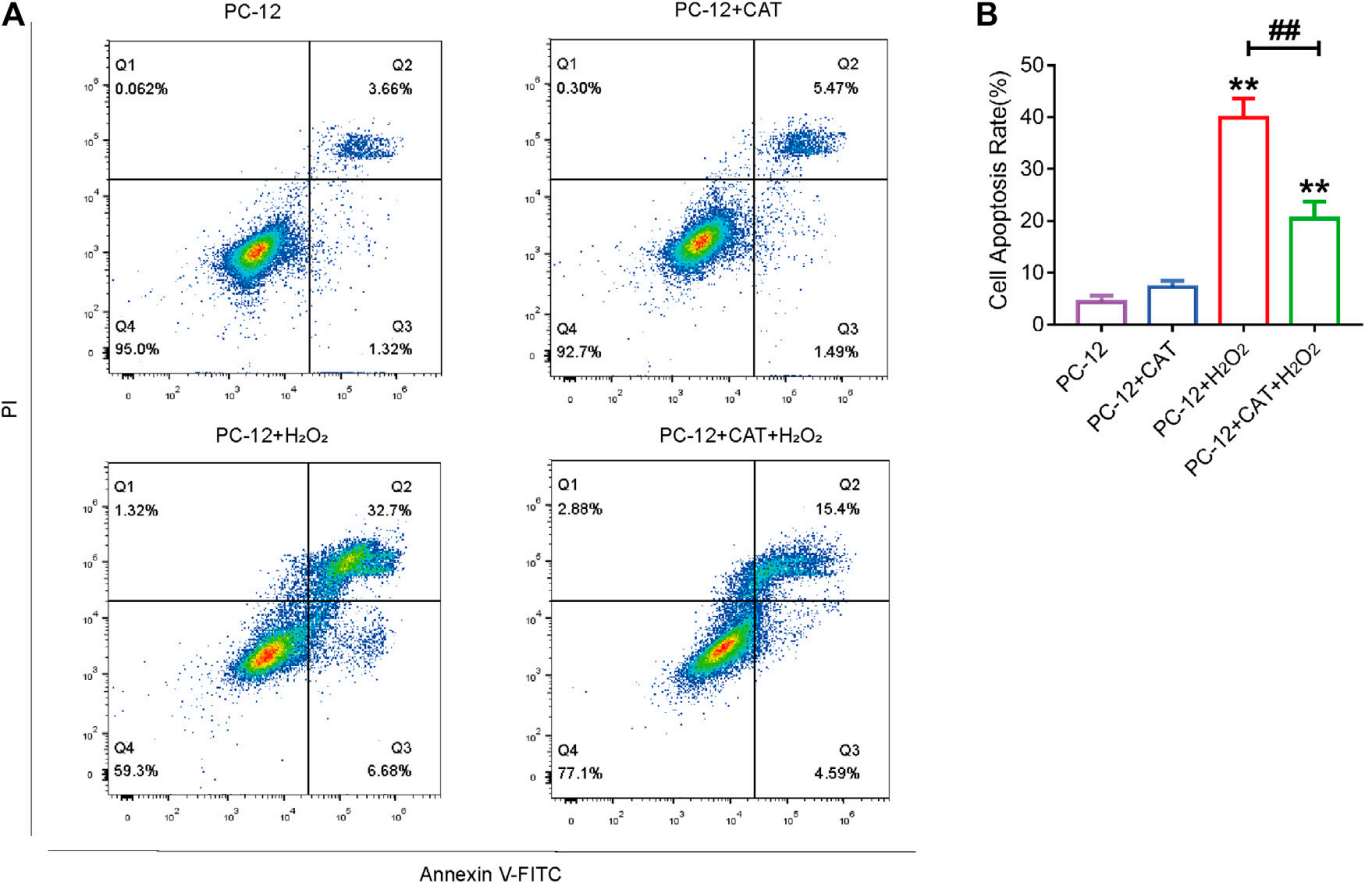
CAT treatment reduces hydrogen peroxide-induced apoptosis in PC-12 cells. **(A)** Annexin V-FITC/PI staining for cell apoptosis analysis. **(B)** Statistical result of apoptosis rate in PC-12 group, PC-12 + CAT group, PC-12 + H_2_O_2_ group, and PC-12 + CA + H_2_O_2_ group. Student’s t test was used to analyze flow cytometry results. “*” represents *p* < 0.05, “**” represents *p* < 0.01 versus the PC-12 group. “##” represents *p* < 0.01 comparing the PC-12 + H_2_O_2_ group to the PC-12 + H_2_O_2_ + CAT group with statistical significance. Data are represented as mean ± SD (*n* = 3).

**FIGURE 8:**
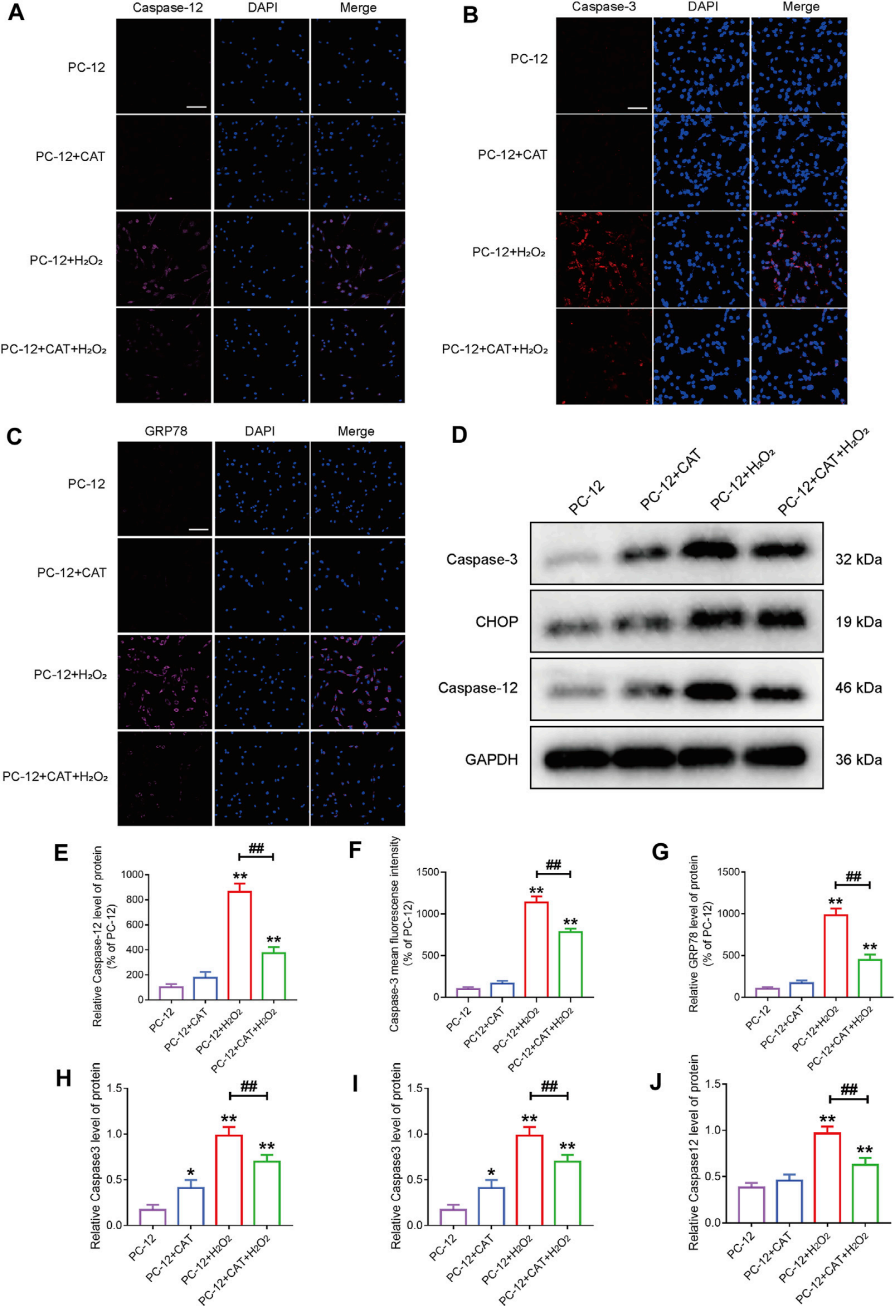
CAT reduces the damage of hydrogen peroxide to PC-12 cells. **(A–C)** Immunofluorescence staining of Caspase-3 (bright red), Caspase-12 (bright pink), and GRP78 (bright pink), in the PC-12 group, PC-12 + CAT group, PC-12 + H_2_O_2_ group, and PC-12 + CAT + H_2_O_2_ group. The nuclei are labeled by DAPI (blue), and magnification is ×40. **(D)** Western blotting analysis showing protein expressions of Caspase-3, CHOP, and Caspase-12. **(E–G)** Caspase-3, Caspase-12, and GRP78 fluorescence intensity (% of PC-12). **(H–J)** Quantitative analysis of the protein expression of Caspase-3, CHOP, and Caspase-12. Student’s t test was used to analyze immunofluorescence staining and Western blotting results. “*” represents *p* < 0.05, “**” represents *p* < 0.01 versus the PC-12 group. “##” represents *p* < 0.01 comparing the PC-12 + H_2_O_2_ group to the PC-12 + H_2_O_2_ + CAT group with statistical significance. Data are represented as mean ± SD (*n* = 3).

Studies have reported that cell morphology was changed and apoptosis was induced after PC-12 cells were exposed to hydrogen peroxide. In this experiment, we hypothesized that pretreatment with CAT would increase cell viability and reverse hydrogen peroxide-induced apoptosis by inhibiting ER stress. According to the results of flow cytometry, we could find that treatment with CAT reduced the hydrogen peroxide-induced apoptosis of PC-12 cells (Figures 7A, B). Indicated by immunofluorescence results, the Caspase-3 expression (red fluorescence), Caspase-12, and GRP78 expression (pink fluorescence) were increased in hydrogen peroxide-treated PC-12 cells, indicating that PC-12 cells underwent apoptosis. By contrast, the Caspase-3, Caspase-12, and GRP78 expression were decreased significantly after CAT treatment, indicating that CAT can protect PC-12 cells from hydrogen oxide-induced apoptosis by inhibiting ER stress (Figures 8A–C, E–G). Similar results were revealed in Western blot results that CHOP, Caspase-12, and Caspase-3 were upregulated in hydrogen peroxide-treated cells, while downregulated after CAT treatment (Figures 8D, H–J). Taken together, our results indicated that CAT can protect PC-12 cells from hydrogen peroxide-induced apoptosis by inhibiting ER stress.

## Discussion

Secondary damage after SCI is a major cause of massive neuronal cell death and difficult recovery of motor function (Li et al., 2020; Jin et al., 2021). Because neural cells have difficult-to-recover properties (Fischer et al., 2020), the most important factor to promote recovery from SCI is to protect neural cells (Xue et al., 2021). In our study, CAT played an important role in promoting recovery from SCI in rats, and after its administration, the locomotor function of hind limbs in SCI rats was recovered. CAT protects neuronal cells by reducing apoptosis *via* inhibiting ER stress and in turn, promotes motor function recovery in SCI.

RG has been used to treat aging and dementia in TCM for a long time (Lee et al., 2011). We hypothesized that RG can act as a protective agent for neurons after SCI. The SCI rats were fed with RG decoction for 30 days, and functional recovery was assessed. It was revealed that the locomotion of the lower limbs was slightly restored in the RG-treated rats when compared with the SCI group. We then screened the constituents in RG based on this phenomenon and further found the excellent drug efficacy of CAT. CAT simultaneously, as a major component of RG, has been reported to display neuroprotective and antiapoptotic effects (Liang et al., 2009; Cai et al., 2011; Liu et al., 2014). In addition, CAT has been found to have anti-inflammatory and neuroprotective roles in inflammation-mediated Parkinson’s disease, which can reduce neuronal degeneration caused by inflammation-triggered oxidative stress and ER stress (Liu et al., 2014; Xiong et al., 2017; Gao et al., 2018). CAT was also reported to protect podocytes in diabetic nephropathy *via* inhibiting apoptosis. It follows that the pharmacological effects of CAT are in good agreement with the situation that a large number of neuronal cells are apoptotic after SCI, but few studies have been reported on the treatment of SCI by CAT; thus, CAT has the potential to become a clinical therapeutic agent for SCI.

ER stress signaling has been shown to promote the mortality of cells in nerve injury diseases (Saraswat Ohri et al., 2021). CHOP plays a key role in ER stress-induced apoptosis (Zinszner et al., 1998), and it is associated with neurodegeneration in Alzheimer’s disease animal models. In an *in vivo* neurotoxin model of Parkinson’s disease, neurons undergo apoptosis and CHOP is activated as a result of perturbations in ER calcium levels (Nakandakari et al., 2019; Hong et al., 2020). After SCI, CHOP expression was inhibited by CAT administration, which is an indicator of ER stress inhibition. Thirty days after SCI, CHOP expression was increased in the remaining white matter, suggesting that CHOP is involved in ER stress-mediated apoptosis. The misfolded proteins accumulated abnormally, causing the Caspase-12/Caspase-3 activation and upregulated expression of GRP78, which subsequently leads to cell apoptosis, after the occurrence of ER stress (Wang et al., 2018; Li et al., 2019b). Our study found that CAT could effectively inhibit the expressions of Caspase-12/Caspase-3 and GRP78 and reduce cell apoptosis in the SCI. After 30 days CAT treatment also inhibited the expressions of Caspase-12 and GRP78 in the remaining white matter, which would contribute to the recovery of motor function. Meanwhile, in the detection of protein level expression, after treatment with CAT, the protein levels related to ER stress were all downregulated and the levels such as apoptosis promoting protein Bax and Caspase3 were inhibited, whereas the level of apoptosis inhibiting protein Bcl-2 was increased. It was further demonstrated that CAT reduced apoptosis by inhibiting Caspase3/Bax/Bcl-2 pathway.

The recovery of neurons is an important indicator to evaluate the recovery of SCI patients. To further verify that CAT reduced neuronal apoptosis by inhibiting ER stress, we detected the neuronal cell indicator. As an axonal membrane protein, GAP43 is involved in the extracellular growth and regeneration of nerve cells *in vivo*. It is abundantly expressed during the regeneration and development of neurons during injury (Zhu et al., 2020b; Kim et al., 2021). MAP-2 contributes to the building of the neuronal cytoskeleton at every stage of the nervous system (Subbarayan et al., 2020; Zhou et al., 2021). After the administration of CAT, the expression of NeuN was increased, which proved that the survival of neurons was improved compared with the SCI group, and the large expression of GAP43 and MAP-2 was also predicted to suggest that neurons were undergoing further recovery, which proved the neuroprotective effect of CAT, and the recovery of hindlimb motor function of rats in behavior also further proved that the protective effect of CAT.

## Conclusion

In this study, we further found that CAT, the main component of Rehmannia decoction, plays a major role in the intragastric administration of rats. Our subsequent study on how CAT can repair SCI showed that CAT can promote functional recovery in SCI rats, as evidenced by improved BBB score and gait imprinting score. Further studies revealed that CAT inhibited ER stress-mediated cell apoptosis, accompanied by increased expression of NeuN, GAP43, and MAP-2, which in turn promotes recovery from SCI. Understanding the roles of TCM components in the pathogenesis of SCI will facilitate the development of TCM as a potential treatment for SCI.

## Data Availability

The raw data supporting the conclusion of this article will be made available by the authors, without undue reservation.
